# Dietary iron and haem iron intake and risk of endometrial cancer: a prospective cohort study

**DOI:** 10.1038/sj.bjc.6604110

**Published:** 2007-12-04

**Authors:** G C Kabat, A B Miller, M Jain, T E Rohan

**Affiliations:** 1 Department of Epidemiology and Population Health, Albert Einstein College of Medicine, 1300 Morris Park Avenue, Room 1301, Bronx, NY 10461, USA; 2 Department of Public Health Sciences, University of Toronto, Toronto, Canada

**Keywords:** endometrial neoplasms, cohort study, dietary iron, haem iron, red meat, body mass index, hormone replacement therapy, metabolic syndrome

## Abstract

We used data from a large cohort study of Canadian women to assess the association of meat intake and dietary intake of iron and haem iron with risk of endometrial cancer. Among 34 148 women with an intact uterus at baseline and followed for a mean of 16.4 years, we identified 426 incident endometrial cancer cases. Data from a food frequency questionnaire administered at baseline were used to calculate intake of all meats, red meat, total dietary iron, iron from meat, haem iron, and non-haem iron. Analyses were carried out using Cox proportional hazards models with adjustment for known risk factors and covariates. We found no association of intake of meat or any of the dietary iron-related variables with risk of endometrial cancer.

Endometrial cancer is largely a malady of affluent, developed societies, showing a more than 10-fold variation between high- and low-incidence countries (Parkin *et al*, 1999). Major risk factors include obesity and oestrogen replacement therapy (Grady *et al*, 1995; Kaaks *et al*, 2002; Persson and Adami, 2002), and recent work suggests that insulin resistance, hyperinsulinemia, and diabetes may also play a role in the disease (Soliman *et al*, 2006; Cust *et al*, 2007; Friberg *et al*, 2007). There is limited evidence that a high intake of red meat may increase risk (Terry *et al*, 2002a; Bandera *et al*, 2007).

A possible association of red meat intake with endometrial cancer risk may reflect a role of haem iron, which derives mainly from red meat and which has greater bioavailability than inorganic iron (Huang, 2003). Free iron is a pro-oxidant and can catalyse lipid peroxidation and DNA damage (McCord, 1998; Huang, 2003). The association of the interrelated conditions of insulin resistance, hyperinsulinemia, and diabetes with endometrial cancer risk also suggests a possible role of excess iron intake or elevated iron stores with endometrial cancer, as these conditions have been linked to excess body iron stores (Tuomainen *et al*, 1997; Fernandez-Real *et al*, 1998; Fernandez-Real *et al*, 2002; Jehn *et al*, 2004). Furthermore, elevated iron stores are associated with increased risk of type II diabetes in healthy women, independent of known risk factors (Jiang *et al*, 2004).

Given that few cohort studies have examined the association of meat intake with risk of endometrial cancer (Zheng *et al*, 1995), and no studies have examined the effects of dietary iron or haem iron intake, we used data from a large cohort study of Canadian women to assess intake of meat, red meat, total iron, and haem iron in relation to endometrial cancer risk. In addition, we explored potential joint effects of iron intake with known risk factors for endometrial cancer.

## MATERIALS AND METHODS

### Study population

The present analysis was conducted in the Canadian National Breast Screening Study (NBSS), a randomised controlled trial of screening for breast cancer, which has been described in detail elsewhere (Miller *et al*, 1992; Terry *et al*, 2002b). In brief, 89 835 women aged 40–59 were recruited from the general Canadian population between 1980 and 1985. On enrollment into the study, information was obtained from participants on demographic, hormonal, and reproductive characteristics, using a self-administered lifestyle questionnaire. Starting in 1982, a self-administered food frequency questionnaire (FFQ), designed to estimate energy and nutrient intake, was distributed to all new attendees at all screening centres and to women returning to the screening centres for rescreening (Jain *et al*, 1982). The FFQ elicited information on usual portion size and consumption of 86 food items and included photographs of portion sizes to assist respondents in quantifying intake. A total of 49 654 dietary questionnaires were returned and were available for analysis. After exclusion of 14 906 women who reported having had a hysterectomy and women whose calorie intake was greater or less than 3 standard deviations from the mean (<730 or >6485 kcal/day), or whose body mass index (BMI) was <15 or >50 kg m^−2^, a total of 34 148 women were available for analysis.

Incident cases of endometrial cancer and deaths from all causes were ascertained, respectively, by means of computerised record linkages to the Canadian Cancer Database and to the National Mortality Database. The linkages to the databases yielded data on cancer incidence and mortality to 31 December, 2000 for women in Ontario, 31 December, 1998 for women in Quebec, and 31 December, 1999 for women in other provinces. For the present analyses, study participants were considered at risk from their date of enrollment until the date of diagnosis of their endometrial cancer, termination of follow-up (the date to which cancer incidence data were available for women in the corresponding province) or death, whichever occurred first. During an average of 16.4 years of follow-up of the dietary cohort, we identified 426 incident endometrial cancer cases.

Data from the FFQ were used to calculate total dietary iron intake using a database described elsewhere (Jain *et al*, 1982). The values for iron intake presented here are for dietary sources alone, because data on iron supplements were not collected. Total intake of meat iron was calculated from the reported intake of 22 meat items and 2 mixed dishes containing meat. Haem iron intake was computed by two methods, using different proportions for haem iron from different types of meat: 69% for beef; 39% for pork ham, bacon, pork-based luncheon meats, and veal; 26% for chicken and fish; and 21% for liver, following Balder *et al* (2006), and, alternatively, using 40% as the average proportion of haem iron in all meats, following Lee *et al* (2004). Results were similar for both methods, and we present data using the first approach. In addition, we assessed risk in association with intake of all meats, red meat, and non-haem iron. All meat- and iron-related variables were calorie-adjusted using the residuals method (Willett and Stampfer, 1986).

### Statistical analysis

Cox proportional hazards models (using age as the time scale) were used to estimate hazard ratios (HR) and 95% confidence intervals (CI) for the association between meat intake and iron intake and endometrial cancer risk. Quintiles of meat- and iron-related variables were constructed based on their distribution in the total population. All multivariate models included the following covariates: BMI (kg m^−2^) (continuous); menopausal status (pre-, peri-, postmenopausal); parity (nulliparous, 1–2, 3–4, 5+ live births); age at menarche (continuous); duration of oral contraceptive use (never, 1–11 months, 12–35 months, 36–71 months, 72+ months); duration of hormone replacement use (never, 1–11 months, 12–59 months, 60–119 months, 120+ months); total caloric intake (continuous); intake of raw vegetables (continuous); alcohol intake (in grams–continuous); physical activity (vigorous, other); and education (three levels). Addition of indicator variables for screening centre (1–15) and randomisation group in the original screening trial (intervention/usual care) did not affect the risk estimates, and therefore, these variables were omitted. Because an effect of iron or haem iron intake could be confounded by intake of other dietary constituents with which they are correlated, we included important food items (red meat, all meat, vegetables, raw vegetables) in additional models. Inclusion of these variables did not affect the risk estimates for iron or haem iron, and as a result they are not included in the final models. To test for trends in risk with increasing levels of the exposures of interest, we assigned the median value for each quintile and then fitted the medians as a continuous variable in the risk models. We then evaluated the statistical significance of the corresponding coefficient using the Wald test (Rothman and Greenland, 1998). In additional analyses, we also treated iron-related variables as continuous variables. As early symptoms of the disease might result in dietary change, we repeated the main analyses, excluding cases diagnosed during the first 3 years of follow-up.

## RESULTS

Cases tended to be older than non-cases, had a higher mean BMI, had lower parity, were more likely to be postmenopausal and to have used hormone replacement therapy, and were less likely to have used oral contraceptives, compared with non-cases (Table 1). Body mass index and duration of hormone replacement use showed dose–response relationships with endometrial cancer risk, and parity and duration of oral contraceptive use were inversely associated with risk (data not shown; see Silvera *et al*, 2005: Table 1). Cases and non-cases were similar in terms of age at first live birth, smoking status (as well as intensity and duration of smoking), and alcohol consumption.

In both age- and multivariate-adjusted models, none of the meat or iron-related variables was associated with risk of endometrial cancer (Table 2). For all variables except non- haem iron, HRs for the highest quintiles of exposure relative to the lowest quintile were below 1.0, but there was no trend over increasing quintiles and none of the point estimates was statistically significant. For non- haem iron intake, some HRs were elevated, but, again, none was statistically significant and there was no clear trend. Similar results were obtained when iron and meat intake were analysed as continuous variables (data not shown). When cases diagnosed during the first 3 years of follow-up were excluded (*n*=53), the patterns were similar to those described above, and no associations or trends were seen with any of the six variables.

There was no association between haem iron intake and endometrial cancer risk within strata of BMI, hormone replacement therapy use (ever or never), or menopausal status (pre- or postmenopausal). The results for meat-related variables and the other iron-related variables were similar (data not shown).

## DISCUSSION

The present analysis showed no association between intake of meat or red meat or dietary intake of iron, haem iron, iron from meat sources, or non- haem iron and risk of endometrial cancer. We also found no association of any of these variables with endometrial cancer risk within strata of BMI or hormone replacement use, the two strongest risk factors in our study, or within strata of menopausal status, which is an important determinant of body iron levels.

The few previous studies of meat and red meat intake in relation to endometrial cancer were mainly case–control in type, while a recent systematic review and meta-analysis has examined the available evidence in detail (Bandera *et al*, 2007). The random effects pooled odds ratio in the meta-analysis of meat intake (7 of the 10 studies had information on all types of meat combined, while the remaining 3 had information on ‘meat, unspecified’) and endometrial cancer was 1.26, 95% CI 1.03–1.54, per 100 g/day increase in intake. However, there was a large degree of heterogeneity between the individual study results. Results of the meta-analysis of red meat intake were somewhat stronger: pooled odds ratio for the seven case–control studies was 1.51, 95% CI 1.19–1.93 per 100 g/day increase in intake.

Only one cohort study of 23 000 postmenopausal women followed for 7 years had adequate numbers of cases (216) to permit a conventional analysis (Zheng *et al*, 1995). Relative risks for both total meat intake and red meat intake were 1.0 and 1.1 for the intermediate and extreme tertiles, respectively, relative to the lowest tertile (no CIs were reported) (Zheng *et al*, 1995). Our results for total meat and red meat intake are consistent with those from this previous cohort study. Inclusion of the results of these two cohort studies in a meta-analysis would be expected to somewhat reduce the summary estimate of the relative risk for red meat intake and endometrial cancer.

No previous studies have examined dietary iron intake in relation to endometrial cancer risk. However, a case–control study from Sweden (Terry *et al*, 2002a) reported that use of iron supplements appeared to increase risk (OR =1.7, 95% CI 0.9–3.3, *P* for trend =0.03). We did not have information on use of iron supplements, which represent an important contributor to total iron intake in users. In a cohort study of New York City women enrolled from 1985 to 1991 (Kato *et al*, 1999), 69% of participants reported use of vitamin/mineral supplements, which accounted for 38% of total iron intake. Other surveys carried out in the United States in the 1980s indicate that over 40% of women currently consumed one or more vitamin or mineral supplements, and fewer than half of users reported taking a preparation containing iron (Stewart *et al*, 1985). We were unable to find similar data pertaining to Canada in the early 1980s, when enrolment in NBSS took place. While iron supplements contribute to total iron intake, most formulations do not contribute to haem iron intake (Frykman *et al*, 1994).

The present study has a number of strengths. We had a relatively large number of incident endometrial cancer cases and were able to control for known risk factors, including BMI, hormone replacement therapy, parity, oral contraceptive use, and physical activity, as well as other covariates, including total caloric intake. In agreement with previous studies, in our data BMI and duration of hormone replacement use showed dose–response relationships with endometrial cancer risk, and parity and duration of oral contraceptive use were inversely associated with risk. Our questionnaire included 22 questions on different types of meat and meat-containing dishes, and we used two different approaches to estimating haem iron intake, both of which yielded similar results. Finally, the results were unchanged when cases diagnosed during the first 3 years of follow-up were excluded from the analysis.

A number of limitations should also be mentioned. Only baseline exposure data were available, and dietary and other exposures may have changed over the 16 year average follow-up period, resulting in some degree of non-differential misclassification of exposure, which would reduce the power to detect an effect of the variables under study. Also, we also did not have information on a personal history of diabetes.

In summary, in this large prospective cohort study, we found no suggestion of an association of intake of meat, red meat, or dietary intake of iron or haem iron assessed at baseline with risk of subsequent endometrial cancer. Furthermore, we found no associations of any of these variables within strata of established risk factors. Future studies should improve on the assessment of iron and haem intake by using repeated measurements and obtaining information on use of iron-containing supplements and a personal history of metabolic syndrome and diabetes.

## Figures and Tables

**Table 1:** Baseline characteristics of the study population by outcome

**Factor**	**Incident endometrial cancer cases (*n*=426)**	**Non-cases (*n*=33 722)**
Mean age at baseline (years)	50.0 (5.4)	48.0 (5.6)
Mean BMI (kg m^−2^)	27.0 (5.9)	24.7 (4.1)
Mean age at first live birth^a^	24.8 (4.8)	24.7 (4.8)
		
Parity (%)		
Nulliparous	21.8	16.1
1–2	36.6	37.0
3–4	34.7	37.7
5+	6.8	9.3
		
Postmenopausal (%)	41.2	33.6
		
Cigarette smoking		
Never	55.6	53.5
Former	29.1	27.7
Current	15.3	18.9
		
Alcohol consumption (%)		
>10 g/day	26.4	27.6
		
Oral contraceptive use (% ever)	50.0	61.1
HRT use (% ever)^b^	45.7	33.0

BMI=body mass index; HRT=hormone replacement therapy.

a Among parous women.

b Results among postmenopausal women only.

**Table 2:** Multivariate-adjusted HR and 95% CI for the association of intake of iron-related variables with risk of incident endometrial cancer

**Factor**	**Cases**	**Person-years**	**Age-adjusted HR (95% CI)**	**Multivariate-adjusted HR^a^ (95% CI)**
*All meat (g/day)*				
<157.71	92	115 817	1.00 (reference)	1.00 (reference)
157.71–<187.83	98	114 520	1.09 (0.82-1.45)	1.11 (0.83–1.48)
187.83–<217.29	70	113 712	0.79 (0.58–1.08)	0.83 (0.60–1.15)
217.29–<257.86	86	110 471	1.00 (0.74–1.34)	0.93 (0.68–1.27)
⩾257.86	78	104 987	0.96 (0.71–1.30)	0.83 (0.60–1.15)
*P for trend*			*0.58*	*0.14*
				
*Red meat (g/day)*				
<48.49	87	113 996	1.00 (reference)	1.00 (reference)
48.49–<66.33	91	113 187	1.07 (0.79–1.43)	1.14 (0.84–1.55)
66.33–<83.47	85	112 695	1.02 (0.75–1.37)	1.08 (0.79–1.47)
83.47–<108.99	95	110 401	1.16 (0.87–1.56)	1.26 (0.93–1.72)
108.99+	64	104 224	0.82 (0.60–1.14)	0.86 (0.61–1.22)
*P for trend*			*0.50*	*0.75*
				
*Total Iron (mg/day)*				
<11.90	82	115 624	1.00 (reference)	1.00 (reference)
11.90–<12.90	104	114 890	1.27 (0.95–1.70)	1.21 (0.90–1.63)
12.90–<13.82	86	113 925	1.05 (0.77–1.42)	1.08 (0.79–1.48)
13.82–<14.99	83	111 752	1.03 (0.76–1.39)	0.95 (0.69–1.32)
⩾14.99	71	104 795	0.93 (0.68–1.28)	0.90 (0.64–1.26)
*P for trend*			*0.31*	*0.22*
				
*Meat iron (mg/day)*				
<3.40	91	116 322	1.00 (reference)	1.00 (reference)
3.40–<4.23	99	114 992	1.11 (0.83–1.47)	1.08 (0.80–1.45)
4.23–<5.02	81	113 035	0.94 (0.70–1.27)	0.94 (0.69–1.29)
5.02–<6.11	86	111 567	1.00 (0.75–1.34)	0.98 (0.72–1.34)
⩾6.11	69	104 084	0.87 (0.64–1.19)	0.79 (0.57–1.11)
*P for trend*			*0.29*	*0.16*
				
*Heme iron (mg/day)*				
<1.58	93	115 820	1.00 (reference)	1.00 (reference)
1.58–<1.99	96	114 575	1.06 (0.80–1.41)	1.10 (0.82–1.48)
1.99–<2.40	86	113 127	0.98 (0.73–1.31)	1.00 (0.74–1.37)
2.40–<2.95	82	111 776	0.94 (0.70–1.27)	0.98 (0.72–1.35)
>2.95	69	104 703	0.85 (0.62–1.16)	0.82 (0.59–1.16)
*P for trend*			*0.22*	*0.22*
				
*Non-heme iron (mg/day)*				
<14.25	76	113 128	1.00 (reference)	1.00 (reference)
14.25–<21.02	99	113 563	1.29 (0.96–1.74)	1.33 (0.97–1.80)
21.02–<28.74	96	114 442	1.23 (0.91–1.66)	1.25 (0.91–1.71)
28.74–40.30	68	112 566	0.87 (0.63–1.21)	0.86 (0.61–1.22)
40.30+	87	107 287	1.16 (0.85–1.57)	1.10 (0.79–1.53)
*P for trend*			*0.76*	*0.50*

CI=confidence interval; HR=hazard ratio. ^a^Adjusted for age (continuous); body mass index (kg m^−2^) (continuous); menopausal status (pre-, peri-, postmenopausal); parity (nulliparous, 1–2, 3–4, 5+ live births); age at menarche (continuous); duration of oral contraceptive use (never, 1–11 months, 12–35 months, 36–71 months, 72+ months); duration of hormone replacement use (never, 1–11 months,12–59 months, 60–119 months, 120+ months); total calorie intake (continuous); intake of raw vegetables (continuous); alcohol intake (in g/day–continuous); physical activity (vigorous, other); and education (three levels).
